# Crossability of miniature rose and quantitative and qualitative traits in hybrids

**DOI:** 10.3389/fpls.2023.1244426

**Published:** 2023-09-05

**Authors:** Ezgi Doğan Meral

**Affiliations:** Department of Horticulture, Bingöl University, Bingöl, Türkiye

**Keywords:** hybridization, pot rose, miniature rose, inheritance, quantitative characters

## Abstract

Potted miniature roses are popular indoor ornamental plants. Due to consumer demands, different varieties are introduced to the market every year. The most widely used method for the development of potted miniatures is cross breeding. Six divergent popular pot miniature roses as female parents and *Rosa centifolia* and Black Baccara as male parents as well as 190 of their F_1_ hybrids were studied to determine the extent of crossability and the heterosis effects for several quantitative and qualitative traits for determining miniature rose breeding potential. The percentage of pollen viability and the pollen germination rate differed between 48.61% and 61.27% and between 23.26% and 32.19%, respectively. All cultivars exhibited a strong correlation between the fruit set, the weight of fruit, the total set, the weight of seed, the number of seeds per fruit, and the seed germination rate. The cultivar Rosa White Star as a female parent exhibited a good fruit set and crossing success, whereas Juanita Kordana showed a poor crossing success rate. The maximum setting after the crossing was that of Rosa White Star × *R. centifolia* comprising 75% of fruits, 132 total seeds, and seed weight of 0.68 g. The highest number of seeds per fruit (12.63) was obtained from Rosa Bling Love Star × *R. centifolia*, and the Red Romance × Black Baccara had the maximum germination of seed rate (48%). The potential for heterobeltiosis and heterosis varied and exhibited a contrasting performance for various qualitative and quantitative traits between F_1_ progenies. The parents and F_1_ hybrids were sorted into three major groups by cluster analysis based on the phenotypic variation.

## 1 Introductıon

Roses have gained the name of “queen of flowers” due to their floral characteristic and plant habits. Roses have been grown and bred to serve for indoor plants, cut flowers, landscaping, and essential oils. Miniatures are a group of rose families that have all the characteristics of a standard rose but are dwarfs in all respect. The roses sold for indoor use are known as “pot rose” that can be used more versatile than other roses. They have different flower types, colors, and forms (Rubert, 1977) and are among the ornamental plants whose demand has increased day by day. Pot roses rank among the top two pot flowers and are worth approximately 14.3 million euros, and 15.7 million pieces of pot miniature roses were sold in worldwide pot flower sales according to Royal FloraHolland (2020) (Flora Cultural International, 2021). The increasing consumer demand for potted miniature roses motivates the breeders into the breeding programs and successfully improves many new miniature rose cultivars.

The development of new rose varieties involves high investment and labor. Therefore, there is a constant need for genetically improved breeding to combat conditions and for scientifically rigorous evaluation of inheritance processes to adequately document rose breeding. Crossing between closely related varieties with common ancestry obtains better results, which is commercially desirable; however, compatibility barriers may limit crossing success (Younis et al., 2014). Other problems include sterility and non-fertility of pollen donor parents. Therefore, breeders desire to integrate fertile parents into gene pools. Flower color, flower size, the number of petals, plant habit, fragrance, the number of flowers per stem, and flower longevity are the primary selection criteria in potted miniature rose breeding. Many rose cultivars have been developed with classical breeding approaches until today. These cultivars certainly ensure the criteria of the breeder for desirable traits, but gene crossing was limited. As a result, less variation in cytology-occurred compatibility decreased among candidate cultivars. Moreover, genetic barriers to hybridization are often an obstacle to obtaining interspecific hybrids in rose (Abdolmohammadi et al., 2014; Dogan et al., 2022). Furthermore, potted miniature roses have not a rich source of pollen for pollinators, and miniatures are known for their difficult sexual reproduction from pollination to seed set and germination (Datta, 2018). Therefore, although some researchers have reported that miniature roses got mini roses when crossed with climber, floribunda, cut flowers, or bush roses (Datta, 2018), in miniature rose breeding, less attention has been paid to scientifically rigorous studies on the quantitative and qualitative traits in their hybrids.

The present study aimed to cross the potential of some miniature rose varieties to compare crossability among the cultivars and the morphology of hybrid progeny.

## 2 Materials and methods

### 2.1 Material

The study was carried out on 25 May 2019 to 30 August 2022 in the rose breeding greenhouse, Department of Horticulture, Faculty of Agriculture, Ankara University (Turkey) (39°57′40.2″N 32°51′51.7″E). In this study, six different tetraploid commercial pot miniature roses (Juanita Kordana, Rosa Shining Star, Red Romance, Orange Romance, Rosa White Star, and Rosa Bling Love Star) were used as female parents, and a *R. hybrida* tetraploid cut rose cultivar (Black Baccara) and *Rosa centifolia* (tetraploid) (Dogan et al., 2020) were used as male parents (**Table 1**).

**Table 1:** Nuclear DNA contents and ploidy levels of the roses in the study (Dogan et al., 2020; Dogan, 2022; Dogan et al., 2023).

Species/Variety	DNA (pg/2C)	Ploidy Level
Rosa Shining Star	2.37	Tetraploid
Red Romance	1.95	Tetraploid
Orange Romance	2.43	Tetraploid
Rosa White Star	2.39	Tetraploid
Rosa Bling Love Star	2.22	Tetraploid
Juanita Kordana	1.97	Tetraploid
*Rosa centifolia*	2.43	Tetraploid
Black Baccara	2.34	Tetraploid

Twenty plants of each genotype were cultivated in single pots with perlite and cocopeat at the Ankara University. During the vegetation period, the temperature inside the greenhouse was kept 23 ± 5°C and the relative humidity between 60-70%. By using a 55% heat-shaded screen, the plants are prevented from being damaged by the intense light in summer. Water and fertilization were provided to the plants by drip irrigation system using a fertigation computer.

### 2.2 Method

#### 2.2.1 Hybridization

Prior to hybridization, pollen parents were tested for pollen viability and germination *in vitro.* Anthers of cultivars were collected from the tight floral buds of 10 plants a day before. The pollen was taken into a glass bottle and kept at 24°C and 60%–65% humidity for 24 h in an incubator for dusting. The pollen viability was examined using the IKI (iodine potassium iodide) test (0.5 g of iodine and 1 g of potassium iodide; Eti, 1991). Pollen grains was spread on a drop of the solution on a coverslip. Four replicates and randomly chosen five fields were counted with a light microscope (×100). *In vitro* pollen germination of the cultivars was assessed using a germination medium (10 ppm (parts per million, (mg/L)) boric acid and 20% sucrose in 1% agar: Eti, 1990) using three petri dishes for each replicate. Four replicates were performed for each two pollen parents. Pollen was placed on the medium with a paintbrush, and, after 24 h of incubation at 24°C and 60%–65% humidity, pollen germination was evaluated. Pollen was assumed to germinate when a pollen tube reached a length of at least 1.5 times the pollen diameter (Leus, 2005; Dogan et al., 2020) with a light microscope (×100), and 250 pollen grains were calculated for each field.

**Figure 1:**
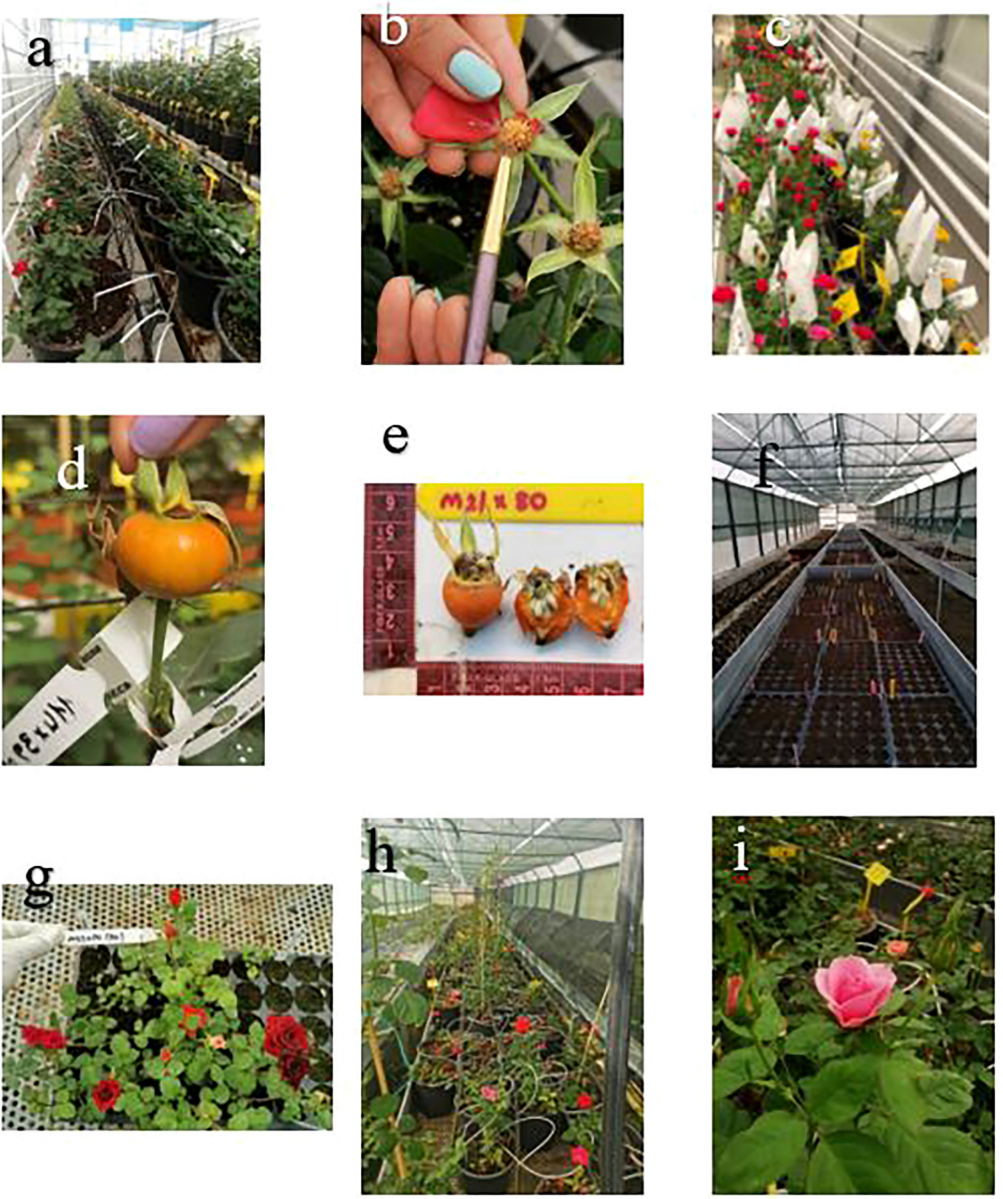
Hybridization to obtaining F_1_ plants. **(A)** Genotype of parent. **(B)** Hybridization. **(C)** Covering the buds with paper bag. **(D)** Mature fruit. **(E)** Removing seeds from the fruit. **(F)** Germination of the seeds. **(G)** Germinating seeds. **(H)** F_1_ genotypes. **(I)** The progeny of Rosa Bling Love Star × *R. centifolia*.

Both pollen and seed parents were prepared for hybridization. Pollen grains were collected and stored in an incubator (22°C and 60%–65% humidity) for dusting, and seed parents were done emasculating when flower buds were in 50%–60% open stage in the morning to avoid self-pollination. Pollen was sprinkled on the stigma of emasculated plants with a brush in the morning, and the flower buds were covered in paper bags. Hybridization was made during the month of July 2019. All crosses were labeled and again covered with taper bags (**Figure 1**).

#### 2.2.2 Data collection

Mature fruits (hips) were harvested in November 2019, and seeds were extracted from pseudo fruit and placed moist in peat at 4°C for 4–12 weeks to break dormancy for germination. After that, seeds were sown in a cocopit and peat mixture. For each cross starting from cross-pollination, data were recorded for the fruit set, total seeds, the number of seeds/fruits, fruit weight, and total seed weight. Seedlings were transplanted into pots and cultivated for 1 year. Progenies were budded in April 2021 for morphological and growth comparisons. From April to November 2022, data on morphological traits were compared in terms of the performance of both progenies and parents.

Full petals were recorded during the full-bloom stage. The flower diameters were measured using a digital caliper during the period of active growth of flowers. The distance from the root of the plant to the tip of the terminal bud was measured, and the plant height was expressed in centimeters. The scent of flowers was also predicted using the following rating scale: 3 = strong fragrance, 2 = mildly scented, and 1 = no discernible fragrance. Color exploitation in flowers is determined and expressed in percentage (%) of progeny. The foliage was also estimated using the following rating scale: 3 = gloss dark green; 2 = gloss light green, and 1 = matte green. Flower persistence day is examined, which is the time from the blooming of the flowers to the day when the plants start to lose their aesthetic appearance (the petals begin to fade and fall). Bush shape was classified as regular or compact, scattering, or open with intermediate rating levels. The scale for assessment of bush shape degrees is as follows: 3 = regular branching, compactness; 2 = less regular branching, but compactness in growth or irregular branching and partial overall compactness; and 1 = highly irregular branching with an open framework. The total number of flowers per stem of progeny (all flowers were counted from the first bloom until the period just prior to pruning) was counted.

#### 2.2.3 Statistical analysis

A randomized plot design with four replications was used to establish the experiment to ascertain pollen viability and germination rates. In each replication, an average of 250 pollen grains were counted in five randomly selected fields. Studies on crossbreeding were conducted using a randomized plot design with four replications. Five blooms were replicated in each cross, resulting in a total of 20 crosses for each combination. Data from pollen quality and crossbreeding studies were analyzed using IBM SPSS Statistics 26 software. Pairwise comparisons between all means were subjected to the Duncan’s multiple range test, and differences were determined using analysis of variance. To define vigor-related effects on the parameters under study, better-parent (BP) and mid-parent (MP) heterosis estimations for the performance of F_1_ progenies were made by the following formula (Fonseca and Patterson, 1968; Nadeem et al., 2015):


$$\text{Ht}\%=\left[\left({\text{F}}_{1}-\text{BP}\right)/\text{BP}\right]\times 100$$

$$\text{Hbt}\%=\left[\left({\text{F}}_{1}-\text{MP}\right)/\text{MP}\right]\times 100$$

## 3 Results

### 3.1 Pollen viability and germination, fruit sets, number of seeds, germination of seed

The Black Baccara and *R. centifolia* pollen were obtained as a bulk of at least 20 flowers (each one collected from a different plant of the same genotype) by gathering the anthers. *R. centifolia* showed the highest pollen viability percentage at 61.27% although the pollen germination rate was 32.19%. The percentage of the pollen viability of Black Baccara was 48.61%, whereas the percentage of pollen germination was 23.26%.

All the values of parameters such as fruit set, weight of fruit, total seed, weight of seed, seed per fruit, and germination of seed showed significant results within all crosses. The maximum percentage of fruit set was observed in crosses of Rosa White Star (65.0%–75.0%) (**Table 2**). A minimum success percentage of the fruit set (20.0%) was observed in the crosses of Juanita Kordana × Black Baccara, Orange Romance × Black Baccara, and Rosa Shining Star × *R. centifolia.* Orange Romance × *R. centifolia* as seed parents showed the highest value of weight of fruit (3.85 g) and weight of seed (0.74 g). The maximum total seed was obtained from the combinations in that Rosa White Star was the seed parent. The maximum number of seeds per fruit was produced by crosses of Rosa Bling Love Star × *R. centifolia* (12.63 seeds) followed by Red Romance × Black Baccara (12.50 seeds) and Orange Romance × *R. centifolia* (11.20 seeds). The maximum success germination of seed percentage was determined by Red Romance × Black Baccara (48.00%) and Orange Romance × *R. centifolia* (31.25%), respectively (**Table 2**).

**Table 2:** Comparison of different parameters in crosses of *Rosa* species.

Combination	FS (%)	WOF (g)		TS		WOS (g)		SPF	GOS (%)
♀ × ♂									
Juanita Kordana × *R. centifolia*	30.0 cd	1.02 d		47.00 de		0.14 de		7.83 b	8.51 de
Juanita Kordana × Black Baccara	20.0 d	0.73 e		19.00 f		0.09 e		4.75 d	4.78 e
Red Romance × *R. centifolia*	35.0 cd	1.51 c		40.00 e		0.15 de		5.71 cd	6.25 de
Red Romance × Black Baccara	40.0 cd	3.20 a		100.00 b		0.49 a		12.50 a	48.00 a
Orange Romance × *R. centifolia*	50.0 bc	3.85 a		112.00 a		0.74 a		11.20 a	31.25 b
Orange Romance × Black Baccara	20.0 d	0.80 e		9.00 g		0.10 de		2.25 e	11.11 d
Rosa White Star × *R. centifolia*	75.0 a	3.10 a		132.00 a		0.68 a		8.80 ab	3.61 e
Rosa White Star × Black Baccara	65.0 ab	3.25 a		128.00 a		0.67 a		9.85 a	7.81 de
Rosa Bling Love Star × *R. centifolia*	40.0 cd	3.04 a		101.00 b		0.36 b		12.63 a	39.60 a
Rosa Bling Love Star × Black Baccara	45.0 c	2.78 a		91.00 c		0.24 c		10.11 a	32.97 b
Rosa Shining Star × *R. centifolia*	20.0 d	0.99 d		20.00 f		0.15 de		5.00 d	5.00 e
Rosa Shining Star × Black Baccara	35.0 cd	1.37 c		52.00 d		0.21 cd		7.43 bc	23.08 c

FS, fruit set; WOF, weight of fruit; TS, total seed; WOS, weight of seed; SPF, seed per fruit; GOS, germination of seed (P ≤ 0.05). The letters indicate the differences between the means (P≤ 0,05).

All the parameters were correlated to forecast the degree of relationship with each other. The maximum value (r = 0.944**) for the positive correlation was recorded between the total seed and the weight of the hip. Although a strong correlation was examined between the total seed and the weight of fruit (r = 0.926**), between the weight of fruit and the weight of seed (r = 0.878**), and between the seed per fruit and the weight of fruit (r = 0.827**), respectively, the weakest correlation (r = 0.099) was examined between the fruit set and germination of seed. The percentage of fruit set had a strong correlation with the total seed (r = 0.654**), the weight of fruit (r = 0.481**), and the weight of seed (r = 0.481**) (**Table 3**).

**Table 3:** Correlation between some different parameters for cross-breeding in *Rosa* species.

Parameters	SPF	FS	WOF	WOS	TS	GOS
SPF	1.000					
**FS**	0.513^**^	1.000				
**WOF**	0.827^**^	0.718^**^	1.000			
**WOS**	0.650^**^	0.820^**^	0.878^**^	1.000		
**TS**	0.782^**^	0.797^**^	0.944^**^	0.926^**^	1.000	
**GOS**	0.757^**^	0.099	0.567^**^	0.227	0.402^*^	1.000

*, Significant (P ≤ 0.05); **, highly significant (P ≤ 0.01). FS, fruit set; WOF, weight of fruit; TS, total seed; WOS, weight of seed; SPF, seed per fruit; GOS, germination of seed.

### 3.2 Plant characteristics

There were significant differences in the height the of plant for the selected parents and progenies. The maximum heights (98 cm and 190 cm) were recorded for Black Baccara and *R. centifolia*, respectively. The excellent heights among F_1_ progenies were determined for Rosa White Star × *R. centifolia* (25.40 cm), followed by Red Romance × Black Baccara (heights of 24.62 cm) (**Table 4**). All potted miniature rose parents have a maximum point for bush shape, although the maximum point of bush shape in F_1_ genotype was that of Orange Romance × *R. centifolia* hybrids (1.57 quality point) (**Tables 3**, **4**). Among the parents, Orange Romance, Rosa White Star, and Rosa Bling Love Star, which are among the potted miniature rose varieties, were observed with the highest score (quality points of 3) in leaf color, whereas Rosa White Star × Black Baccara hybrid was observed among the F_1_ genotypes.

**Table 4:** Morphological traits and flower characteristics of parents.

Cultivars/Species	NOPS	NOA	FD (cm)	HOP (cm)	NOPF	FRAG	BS	LC	FC
Juanita Kordana (P1)	54.0 cd	67.0 cd	4.5 d	27.5 b	67.0 b	1.0 a	3.0 a	2.0 a	Red
Red Romance (P2)	58.0 c	72.0 d	6.9 ab	25.5 b	36.0 d	1.0 a	3.0 a	2.0 a	Red
Orange Romance (P3)	80.0 b	139.0 c	7.2 a	26.5 b	25.0 e	2.0 a	3.0 a	3.0 a	Orange
Rosa White Star (P4)	65.0 c	65.0 d	6.6 b	25.1 b	32.0 d	1.0 a	3.0 a	3.0 a	White
Rosa Bling Love Star (P5)	46.7 d	218.0 a	6.0 c	27.5 b	45.0 c	1.0 a	3.0 a	3.0 a	Pink
Rosa Shining Star (P6)	62.7 cd	106.0 c	6.5 b	26.7 b	32.0 d	1.0 a	3.0 a	2.0 a	Yellow
Black Baccara (P7)	46.0 d	98.0 b	10.1 a	127.7 a	45.0 c	2.0 a	2.0 a	3.0 a	Dark Red
*Rosa centifolia* (P8)	160.0 a	190.0 ab	12.5 a	138.5 a	95.0 a	3.0 a	2.0 a	2.0 a	Pink

NOPS, number of pistil per flower; NOA, number of anthers per flower; FD, flower diameter; HOP, height of plant; NOPF, number of petal per flower; FRAG, fragrance; LC, leaf color; BS, bush shape; FC, flower color (P ≤ 0.05). The letters indicate the differences between the means (P≤ 0,05).

### 3.3 Flower characteristic

Significant differences (p ≤ 0.05) were exhibited by the parent genotypes and F_1_ hybrid progenies for flower diameters. Whereas the largest flower diameter was recorded in *R. centifolia* (12.5 cm) from the parents, the largest average flower diameter in F1 genotypes was determined in the Rosa White Star × *R. centifolia* hybrid (6.92 cm). The number of petals varied between 25.0 (Orange Romance) and 95.0 (*R. centifolia*) among the parents; meanwhile, petals varied between 23.33 (Juanita Kordana × Black Baccara) and 39.39 (Rosa Bling Love Star × Black Baccara) among the F_1_ genotypes. The presence or absence of fragrance was changed between quality points of 1 (all miniatures except for Orange Romance) and 3 (*R. centifolia*) among the parents when fragrance quality points changed from 1.0 to 2.0 points among the F1 genotypes. Among the F_1_ genotypes, the number of flowers per plant ranged from 1.50 (Juanita Kordana × *R. centifolia*) to 2.80 (Rosa White Star × *R. centifolia*) (**Tables 4**, **5**). The progeny of Rosa Shining Star × Black Baccara (12.25) exhibited the maximum flower persistence life, followed progeny of Red Romance × Black Baccara (12.20) (**Table 4**).

**Table 5:** Morphological traits and flower characteristics of F1 progenies.

Combination	FD (cm)	HOP (cm)	NOPF	FRAG	BS	LC	NOF	FPL
P1 × P8 (O1)	3.46 c	20.00 d	35.38 a	1.00 b	1.25 a	2.00 a	1.50 a	8.25 b
P1 × P7 (O2)	6.66 a	23.80 a	23.33 d	2.00 a	1.00 a	2.00 a	2.00 a	10.00 b
P2 × P8 (O3)	4.01 c	25.50 a	32.11 a	1.33 ab	1.33 a	2.33 a	2.45 a	9.50 b
P2 × P7 (O4)	5.04 bc	26.62 a	33.77 a	1.00 b	1.21 a	2.19 a	2.27 a	12.20 a
P3 × P8 (O5)	4.82 bc	24.82 a	34.21 a	1.27 ab	1.57 a	2.41 a	1.95 a	9.15 b
P3 × P7 (O6)	3.90 c	25.80 a	27.00 c	1.00 b	1.00 a	2.00 a	2.00 a	9.25 b
P4 × P8 (O7)	6.92 a	25.40 a	28.43 bc	1.60 ab	1.00 a	2.00 a	2.80 a	9.33 b
P4 × P7 (O8)	5.08 bc	23.15 ab	29.00 b	1.00 b	1.40 a	2.70 a	1.70 a	9.45 b
P5 × P8 (O9)	5.71 ab	21.30 cd	33.79 a	1.23 ab	1.25 a	2.63 a	2.73 a	9.15 b
P5 × P7 (O10)	4.12 bc	20.93 cd	39.39 a	1.00 b	1.15 a	2.45 a	2.52 a	10.15 b
P6 × P8 (O11)	4.00 c	21.95 bc	35.42 a	1.00 b	1.00 a	2.00 a	2.00 a	9.00 b
P6 × P7 (O12)	4.30 bc	24.15 a	32.15 a	1.00 b	1.33 a	2.56 a	2.25 a	12.25 a

FD, flower diameter; HOP, height of plant; NOPF, number of petal per flower; FRAG, fragrance; BS, bush shape; LC, leaf color; NOF, number of flower; FPL, flower persistence life (P ≤ 0.05). The letters indicate the differences between the means (P≤ 0,05).

Exploitation in the F_1_ progenies of the petal color was categorized as pink, red, orange, white, and yellow. F_1_ progenies of different colored parents had 61.69% red, 31.84% pink, 3.98% orange, 1.49% white, and 1.00% yellow petals. Juanita Kordana × Black Baccara and Red Romance × Black Baccara combinations, in which both seed parent and pollen parents had red colored petals (70.83%–100%), were highly dominant in the red color among F_1_ plants. Among the F_1_ plants obtained from the combinations of parents with red and pink color petals (Juanita Kordana × *R. centifolia*, Red Romance × *R. centifolia*, Rosa Bling Love Star × Black Baccara, and Rosa Shining Star × Black Baccara), it was determined that the pink color was dominant to the red color. In F_1_ progenies from the combination in which at least one of the parents was red (Orange Romance × Black Baccara, Rosa White Star × Black Baccara, Rosa Shining Star × Black Baccara), the color red was dominant to white, orange, and yellow colors. In F_1_ plants from the combination in which at least one of the parents was pink (Orange Romance × *R. centifolia*, Rosa White Star × *R. centifolia*, and Rosa Shining Star *× R. centifolia*), the pink color was dominant to other color petals (**Table 6**).

**Table 6:** Exploitation in the F_1_ progenies of the petal color.

Progenies	Red (%)	Pink (%)	Orange (%)	White (%)	Yellow (%)
Juanita Kordana × *R. centifolia* (O1)	25.00	75.00	–	–	–
Juanita Kordana × Black Baccara (O2)	100.00	–	–	–	–
Red Romance × *R. centifolia* (O3*)*	33.33	66.67	–	–	–
Red Romance × Black Baccara (O4)	70.83	29.17	–	–	–
Orange Romance × *R. centifolia* (O5)	–	78.38	21.62	–	–
Orange Romance × Black Baccara (O6)	100.00	–	–	–	–
Rosa White Star × *R. centifolia* (O7)	–	100.00	–	–	–
Rosa White Star × Black Baccara (O8)	40.00	30.00	–	30.00	–
Rosa Bling Love Star × *R. centifolia* (O9)	–	100.00	–	–	–
Rosa Bling Love Star × Black Baccara (O10)	36.36	63.64	–	–	–
Rosa Shining Star × *R. centifolia* (O11)	–	100.00	–	–	–
Rosa Shining Star × Black Baccara (O12)	55.56	33.33	–	–	11.11
*Total	**61.69**	**31.84**	**3.98**	**1.49**	**1.00**

* Color average of all F_1_ plants.

Among the parameters, heterosis (Ht) and heterobeltiosis (Hbt) values varied. The heterosis was evident in 33.33% of the hybrid progeny of Juanita Kordana × Black Baccara (O2), for fragrance. The heterosis and heterobeltiosis were evident in 16.50% of the hybrid progeny of Red Romance × *R. centifolia* (O3), for leaf color (**Table 7**).

**Table 7:** Heterosis and heterobeltiosis of F_1_ hybrids for some parameters.

Progenies		FD	HOP	NOPF	FRAG	BS	LC
P1 × P8 (O1)	Ht (%)	−59.29	−78.31	−56.32	−50.00	−50.00	0.00
	Hbt (%)	−72.32	−87.00	−62.76	−50.00	−58.33	0.00
P1 × P7 (O2)	Ht (%)	−8.77	−71.54	−42.40	33.33	−60.00	−20.00
	Hbt (%)	−34.06	−82.93	−65.18	−33.33	−66.67	−33.33
P2 × P8 (O3)	Ht (%)	−58.66	−71.34	−50.98	−33.50	−46.80	16.50
	Hbt (%)	−67.92	−83.03	−66.20	−33.50	−55.67	16.50
P2 × P7 (O4)	Ht (%)	−40.71	−67.86	−16.62	−33.33	−51.60	−12.40
	Hbt (%)	−50.10	−80.72	−24.96	−50.00	−59.67	−27.00
P3 × P8 (O5)	Ht (%)	−51.07	−72.34	−42.98	−49.20	−37.20	−3.60
	Hbt (%)	−61.44	−83.52	−63.99	−57.67	−47.67	−19.67
P3 × P7 (O6)	Ht (%)	−54.91	−69.13	−22.86	−50.00	−60.00	−33.33
	Hbt (%)	−61.39	−81.36	−40.00	−50.00	−66.67	−33.33
P4 × P8 (O7)	Ht (%)	−27.54	−68.95	−55.23	−20.00	−60.00	−20.00
	Hbt (%)	−44.64	−81.66	−70.07	−46.67	−50.00	−33.33
P4 × P7 (O8)	Ht (%)	−39.16	−72.32	−24.68	−33.33	−44.00	−10.00
	Hbt (%)	−49.70	−83.44	−35.56	−50.00	−30.00	−10.00
P5 × P8 (O9)	Ht (%)	−38.27	−76.75	−51.73	−38.50	−50.00	5.20
	Hbt (%)	−54.32	−86.06	−64.43	−59.00	−58.33	−12.33
P5 × P7 (O10)	Ht (%)	−48.82	−75.61	−12.47	−33.33	−54.00	−18.33
	Hbt (%)	−59.21	−85.18	−12.47	−50.00	−61.67	−18.33
P6 × P8 (O11)	Ht (%)	−57.89	−75.85	−44.22	−50.00	−60.00	0.00
	Hbt (%)	−68.00	−85.60	−62.72	−66.67	−66.67	−33.33
P6 × P7 (O12)	Ht (%)	−48.19	−71.31	−16.49	−33.33	−46.80	2.40
	Hbt (%)	−57.43	−82.65	−28.56	−50.00	−55.67	−14.67

FD, flower diameter; HOP, height of plant; NOPF, number of petal per flower; FRAG, fragrance; BS, bush shape; LC, leaf color; Ht, heterosis; Hbt, heterobeltiosis.

### 3.4 Cluster analysis

**Figure 2:**
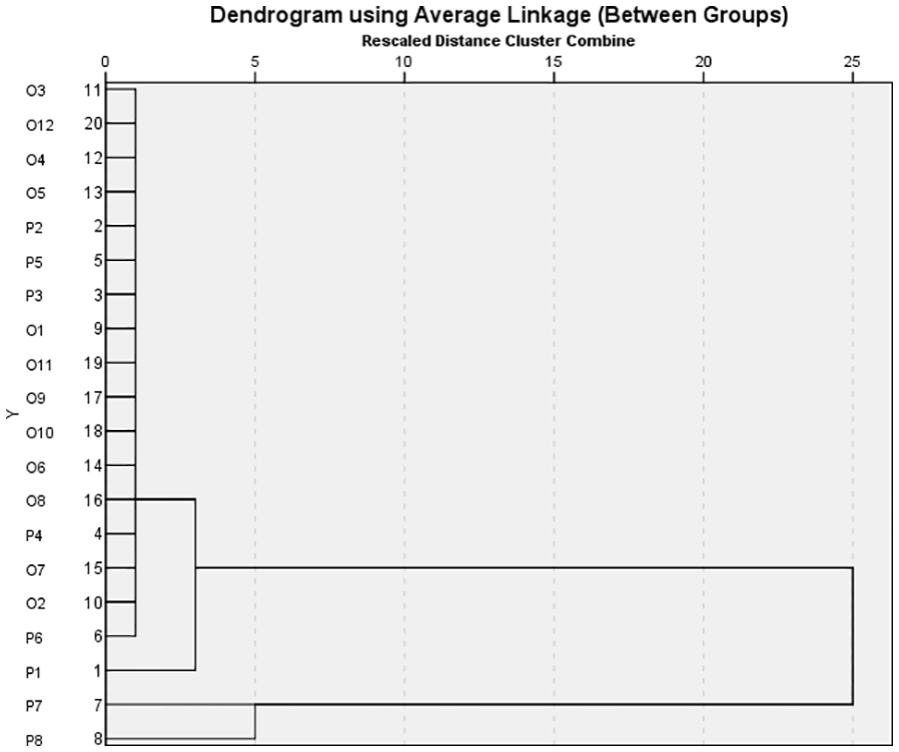
The similarity dendrogram obtained as a result of the clustering analysis carried out considering the morphological characteristics of the rose parents and progenies.

The data obtained as a result of the morphological characterization were evaluated by cluster analysis into three main clusters to reveal the similarity relationships of the genotypes. Most parents were grouped together with their progenies in a clustering method based on phenotypic (quantitative) characteristics (**Figure 2**). In general, miniature pot roses and progenies were in the same group.

## 4 Discussion

In breeding programs, the development of new rose varieties follows a sequence of processes such as fertilization, fruit set, seed formation, germination of seeds, and hybrid progeny after controlled pollination (Nadeem et al., 2013; Dogan et al., 2020). One of the most important steps in crossbreeding is the inclusion of fertile species and varieties in breeding programs. It is very important that the pollinator pollen, which is used as a male parent, is highly viable and germinating. In the studies carried out, the viability rates of some modern roses were found between 17.71% and 52.14% by Dogan (2022), and Pipino et al. (2011) reported that it ranged from 0% to 46.5%. The pollen germination rates of different hybrid tea roses were determined between 0% and 46.5% by Pipino et al. (2011), and Dogan et al. (2020) found that it varies between 24.61% and 45.24%. These studies show similarities with the present study. Although the fruit set, the number of seeds per fruit, and the seed germination rate of miniature roses are generally low, the miniature used as pollen parents and crossed with climbers, floribundas, and shrub roses is recommended (Datta, 2018; Dogan, 2022). In the present study, fertility of the parent cultivars ranged from low to high, most likely caused of multiple genetic factors, mitotic abnormalities, including lethal genes and their deleterious effects, incompatibility of gametes, the heterozygous polyploid, and interspecific factors of parent cultivars (Ogilvie et al., 1991). Overall, there was a satisfactory success rate of 39.58% among all possible crosses, which coincides with the findings of Dogan (2022) and Uran (2022) who observed that 30.3% and 37.92%, respectively, of all miniature crosses develop into mature hips, and the resulting seeds exhibit a germination rate of 18.49%. Cross-incompatibility and disparities at the ploidy level could cause low hip-set percentages in most tetraploid roses. In this regard, the present study is consistent with the findings of other researchers (Morey, 1959; Rajapakse et al., 2001; Kazaz et al., 2020; Dogan et al., 2022). Fagerlind (1954) reported that, although the union of gametes from both parents could occur, not all hybrids might necessarily have a good seed set or viable embryos. Otherwise, environmental conditions (daylight and daily temperature) might affect the seed set and the germination percentage (Gudin et al., 1990). Some growth inhibitors may develop in rose seeds that block seed germination. These inhibitors include abscisic acid in the pericarp and testa, which are physiological barriers in the embryo (Bo et al., 1993; Zhou et al., 2009). Some research studies have reported that hybrid tea roses have a low (below 50%) and non-uniform germination rate (Gudin, 2003; Ueda, 2003; Zlesak, 2005; Anderson and Byrne, 2007). In potted miniature rose breeding, the seed set of miniature roses is generally low. The situation is that the dwarf factor causes infertility in females (Datta, 2018). It has been reported that miniature roses produce few fruits and seeds per fruit when used as the female parent in breeding programs, and the seed germination rate is low. The reason why the seed formation rate of miniature roses is generally low in potted miniature rose breeding may be due to the fact that the dwarfness causes infertility in females. On the other hand, Datta (2018) stated that the fertility of miniature roses is high when used as pollen parents, and a good fruit formation, the number of seeds per fruit, and the germination rate can be obtained when hybridized with climbers, floribundas, or shrub roses. In addition, although many researchers reported that the difference in the number of seeds per fruit was due to different pollen viability and germination rates of the parents, it was reported that wild and old garden roses had a higher pollen quality than hybrid roses, which increased fruit formation and the number of seeds per fruit (Ueda and Hirata, 1989; Gudin et al., 1991; Zlesak et al., 2007; Kazaz et al., 2020). Love et al. (2016) reported that especially the compatibility of the parents affects the success of crossbreeding. In addition, studies have reported that high fruit sets and seed numbers are obtained in parallel with the increase in the amount of pollen applied to the stigma (Falque et al., 1995). Atram et al. (2015) determined that stigma diameter and fruit-bearing capacity affect especially the fruit set and the average number per fruit in roses.

These findings suggest that even species or varieties with a high female crossability rate may not always exhibit great seed efficiency. These many parent–offspring connections and pairings highlight the significance of pollen fertility. However, as a female parent, in miniature roses, the fertility affected the crossing success as much as pollen fertility. Considering that miniature roses with small flower diameters may have less stigma, they had lower seed formation than other modern roses. It has been reported that fruit formation, the number of seeds per fruit, and seed formation are lower in interspecies crosses than that in intraspecies crosses (Kılıç, 2023). Successful results were obtained in fruit formation, the number of seeds per fruit, and seed formation by crossbreeding between species in miniature roses with limited fertility. Considering that seed formation is negligible from hybridization of miniature roses with miniature roses, it can be said that seed formation is related to cross-compatibility rather than the stigma number and pollen germination rates. Both the germination rates of seeds obtained from the combinations of *R. centifolia* and those of Black Baccara did not differ from one another. However, the embryo rescue procedure will improve the likelihood of producing additional hybrids if the old garden roses have post-pollination barriers. The study showed the crossing success of the miniature roses as seed parents. Moreover, there were limited studies in miniature rose breeding. It is thought that this parameter is important for evaluating breeding studies. The inbreeding tendencies of new cultivars could be derived from a small number of productive genotypes with above-average progeny (De Vries and Dubois, 1996). Treatment results in inbreeding depression due to increased homozygosity in the resulting lines and consistency of lethal alleles (Ogilvie et al., 1991). Seedling growth took only 8 weeks to complete vegetative growth and flowering thereafter. Miniature rose progenies (F_1_) of set flowers on completion of juvenility last for 4 to 5 weeks. According to the study, there was a significant association between the weight of the fruit and the quantity of seeds present in each fruit. This is to be expected because the fruit will be heavier with more seeds. Although less strong, there was still a significant relationship between the fruit set and seed germination. This shows that the fruit set and germination have a genetic component but shows that other factors, such environmental conditions, also play a role.

Some parameters in qualitative and quantitative traits were evaluated in parents and hybrid cultivars. These features were evaluated by statistical analysis and the creation of a dendrogram for various qualitative and quantitative characters. Some features have improved positively achieving the purpose of the study, whereas some features remained unchanged. Using the dendrogram approach given in the current study, yield characteristics of some rose varieties were investigated in terms of morphological characteristics and other quantitative characteristics and get similar findings to found in progenies (Nadeem et al., 2015). The plant heights of the F_1_ plants obtained in the combinations showed variation. Although the pollen parents in these combinations were tall, the reason why the F_1_ plants remained between 20 cm and 26.62 cm might be due to the dominant effect of the dwarf gene. Indeed, it has been reported that a dwarf gene locus is controlled by a dominant (*D*) allele gene (Debener, 1999) and, when crossed with miniature, 90% of progeny are expected to be miniature (Datta, 2018). In addition, Byrne (2009) determined that additive genes are effective in terms of plant height that is inherited by polygenic genes. Although the data obtained in the present study in terms of flower diameter are generally similar to the flower diameters of commercial potted miniature roses, the differences in the lower and upper values of the flower diameter size may be caused by the climatic conditions in which the plants are grown, the pot size, the growing environment, the plant growth regulator application, and the differences in cultural processes. In addition, flower diameter is directly related to the number of petals, and the number of petals is controlled by dominant genes (Morey, 1959; Zlesak, 2007). In the present study quantity of petals were double flowers in all roses. Double flowers were all inherited through a single dominant allele, and additive genes stated the number of petal in double genotype (Debener, 2003). The data also reveal that the F_1_ hybrids and their parents had significantly different heights of the plants, flower diameters, and number of petals. The F_1_ hybrids had more petals and greater flower diameters than their parents, and they were often taller than their parents. This shows that the F_1_ hybrids have heterosis or hybrid vigor. Some of the F_1_ hybrids had longer flower persistence life than the parents, whereas others had shorter flower persistence life. Overall, the F1 hybrids in the study had a number of desirable traits, such as larger flower diameters and more petals. These traits are likely due to heterosis or hybrid vigor. However, the data also suggest that the flower persistence life is a more complex trait that is influenced by multiple genes.

Rose colors are due to the presence of pigments like anthocyanin, carotenoids, and flavonols. The anthocyanidins (peonidin, pelargonidin, and cyanidin) ensure a typical color, specially red-colored or purple-colored flowers. The flower color is commonly due to additive gene action of several genes or different kinds of pigments. Rose color has a complex inheritance due to the additive gene action. For instance, the inheritance of the pink flower of *R. multiflora* is controlled by one or two complementary genes. Whereas the pink color is dominant to the red or dark red color, similarly, the light yellow is dominant to the dark yellow and white color is recessive to light yellow and cream (Lammerts, 1945). Debener and Mattiesch (1999) obtained F_1_ plants with 22.12% white and 77.88% pink petal leaves by crossing two different pink rose genotypes and also determined that pink color may have monogenic or oligogenic inheritance. In a study conducted by crossing pink and white rose genotypes, it was determined that 31.58% of F_1_ genotypes had white petals and 68.42% had pink petals (Shupert, 2005). These study findings were consistent with those in the present study.

The fact that F_1_ progeny often has a similar height to the phenotypic of the main parent can be considered an important component in the production of this characteristic, and the use of tiny roses as the seed parent in miniature rose breeding plays an important part in parent selection. The use of *R. centifolia* as pollen parent resulted in obtaining fragrant rose genotypes. Genotypes with large buds were generated by using the Black Baccara variety, which is one of the cut rose varieties, and the use of cut roses in miniature rose breeding as parents can be provided to the offspring in terms of attractive flower color and leaf brightness.

## 5 Conclusion

Breeding studies require a long time and more labor and are costly. The development period for potted miniature roses is 4–5 years; therefore, the characteristics and fertility situations of parents are very important in the selection of hybrid combinations. The present study shows high rates of fruit, many seeds per fruit, and a low germination ratio, a few progeny were obtained while these genotypes had dwarf and large flower diameter plants. In the combinations where *R. centifolia* was used as a pollen parent, fragrant plants get the result that Rose Bling Love Star and Rose White Star have the potential to be utilized as a seed parent, as evidenced by the high fruit set and the number of seeds per fruit. Moreover, qualitative and quantitative of the progenies and calculations of heterosis and heterobeltiosis had proven to be useful tools in evaluating the performance of the offspring on the parents, thus facilitating the selection of cultivar candidates with better-performing traits. Because of the fact that cross-combinations can be determined using the information on parental performance that is now accessible, this study is assumed to help with the breeding success of the miniature roses. In comparison with a random decision, the combinations chosen in this manner are more likely to be successful. Breeders can swiftly adopt the practice of choosing their cross-combinations and parents more carefully to increase the efficacy of their breeding programs and offer more new types to their market.

## Data availability statement

The original contributions presented in the study are included in the article/supplementary material. Further inquiries can be directed to the corresponding author.

## Author contributions

The author confirms being the sole contributor of this work and has approved it for publication.
